# Effects of guanidinoacetic acid on gene expression in adipose tissue of broiler chickens: a differential expression analysis based on small RNA sequencing

**DOI:** 10.3389/fvets.2025.1732976

**Published:** 2026-01-29

**Authors:** Mengqian Liu, Jinrui Ruan, Yu Yang, Mengyuan Li, Zifu Gu, Changrong Ge, Weina Cao

**Affiliations:** 1 College of Animal Science and Technology, Yunnan Agricultural University, Kunming, China; 2 Key Laboratory of Feed Biotechnology, Ministry of Agriculture and Rural Affairs, Institute of Feed Research, Chinese Academy of Agricultural Sciences, Beijing, China; 3 College of Animal Science and Technology, Gansu Agricultural University, Gansu, China; 4 Yunnan Provincial Key Laboratory of Animal Nutrition and Feed, Yunnan Agricultural University, Kunming, China

**Keywords:** adipose, broiler, fat deposition, guanidinoacetic acid, microRNA, transcriptome

## Abstract

This study aims to elucidate the mechanism by which guanidinoacetic acid (GAA) reduces abdominal fat deposition in broilers, focusing on the regulatory role of microRNAs (miRNAs). Excessive fat accumulation in the abdomen is a common issue in meat-type chickens as it reduces feed efficiency and compromises meat quality. By performing small RNA (sRNA) sequencing and analysis on broilers from GAA-treated and control groups, a total of 46 differentially expressed miRNAs were identified. Gene Ontology (GO) and Kyoto Encyclopedia of Genes and Genomes (KEGG) enrichment analyses indicated that these miRNAs were significantly enriched in pathways related to lipid metabolism. Further investigation revealed that GAA likely reduces fat deposition and promotes lipid oxidation by up-regulating gga-miR-103-3p and gga-miR-107-3p, thereby inhibiting their target genes, stearoyl-CoA desaturase (SCD) and transmembrane protein 35B (TMEM35B). These findings provide a theoretical foundation for the application of GAA to improve growth performance and meat quality in broilers.

## 1 Introduction

New intensive farming methods and selective breeding have improved the growth rates and feed conversion efficiencies of broiler chickens (1, 2). Nevertheless, rapid growth in some breeds is associated with increased deposition of fat, particularly in the abdominal and subcutaneous regions (3). A surplus of fat damages meat quality, raises feed costs, impacts the environment and harms consumer health (4–6). Abdominal adipocytes exhibit higher metabolic activity and greater lipogenic potential (7, 8). Therefore, understanding the molecular pathways responsible for abdominal fat accumulation is crucial.

Guanidinoacetic acid (GAA) is the primary biosynthetic precursor of creatine in vertebrates (9, 10). Creatine is nitrogenous organic acid vital for supplying energy for fast growth and development of muscle tissue via the creatine and phosphocreatine pathway (11–13). Studies show that dietary GAA elevates the concentration of creatine in animal tissues to improve performance, feed conversion efficiency, and enhance antioxidant capacity (12, 14). GAA improves the efficacy of utilizing phosphocreatine-derived energy, a speedy source of ATP for muscle and liver tissues, and also aids its synthesis (11, 15–17). Furthermore, studies indicate that creatine, targeting the PI3K signaling pathway, can block the differentiation of adipocyte at the early stage (18). Thermogenic adipocytes may use creatine to activate futile cycles, contributing to the regulation of energy metabolism.

Molecules like stearoyl-CoA desaturase 1 (SCD1) and transmembrane protein (TMEM) family members (e.g., TMEM35B) are already known to modulate lipid synthesis, adipocyte differentiation and energy metabolism. SCD1 acts as a rate-limiting enzyme for monounsaturated fatty acid production and modulates adipocyte lipid composition and metabolic state directly (19, 20). MicroRNA-103/107 (miR-103/107) family also is critical to metabolism because it targets the transcription factor C/EBPα to inhibit adipogenesis and enhances insulin sensitivity (21). It is, however, unclear whether and how the feed additive GAA influences abdominal fat deposition in poultry through these pathways. Studies on the fat-reducing effects of GAA have focused on growth performance, slaughter traits, and a limited set of serum biochemical markers (22, 23), but have not employed systematic transcriptomic or post-transcriptional network analyses. Thus, elucidating how GAA regulates key molecules such as SCD1, TMEM proteins, and miR-103/107 may provide a strategy to mitigate abdominal fat deposition.

Studies on GAA as a poultry feed additive have focused primarily on production performance and meat quality (24–26). In contrast, its specific impact on abdominal fat deposition remains poorly understood, with only limited research, such as the study by Wu et al. (23), having addressed this aspect. Our previous study showed that dietary supplementation with 1.2 g/kg GAA significantly reduced abdominal fat percentage and adipocyte size in Cobb broilers. While both 1.2 and 3.6 g/kg GAA improved growth performance, the lower dose (1.2 g/kg) proved superior for enhancing breast yield, thigh yield, and meat quality (27). Therefore, the 1.2 g/kg dose was selected for further investigation based on its optimal effects on carcass yield and meat quality. However, the mechanism underlying its fat-reducing action remained unknown. To address this, we performed an integrated analysis of microRNA (miRNA) and mRNA expression using bioinformatics, constructing a regulatory network of miRNAs involved in abdominal fat development in Cobb broilers. This work expands the repertoire of known avian miRNAs in abdominal adipose tissue and offers new insights into miRNA-mediated regulation of fat deposition.

## 2 Materials and methods

### 2.1 Ethics statement

All experimental procedures and operations received approval from the Life Sciences Ethics Committee at Yunnan Agricultural University (Approval ID: 202203094). The research was carried out in compliance with regional laws and institutional guidelines.

### 2.2 Collection of abdominal fat tissue from experimental chickens

Hunan Shuncheng Industrial Co., Ltd. supplied one-day-old Cobb chicks, which were then accommodated in stacked cages. Controlled conditions as to temperature, humidity and air flow were maintained in these cages. The diet plan consisted of corn-soybean meal pellets while the control group got the feed. The nutritional composition and makeup of the base diet utilized during the preliminary phase of the study are illustrated in Table 1. At the same time, Table 2 outlines the nutritional characteristics and composition of the base diet implemented in the later phase. All experimental groups received an isocaloric basal diet. The experimental group (GAA group) was supplemented with 1.2 g/kg of guanidinoacetic acid by replacing an equal amount of carrier to maintain identical total caloric content with the control group. Moreover, in the GAA group, we incorporated another 1.2 g/kg dose of guanidinoacetic acid in the basal diet, which was provided to the chickens until they reached 42 days of age. The experiment incorporated solely male broiler chicks. At the end of the experiment, all chickens were humanely euthanized by cervical dislocation. Tissues of abdominal fat of three chickens from each group were collected. The tissues were quickly frozen with liquid nitrogen and stored at −80 °C for analysis.

**Table 1:** Composition of the basal diet (%).

**Ingredients**	**Brooding period**	**Breeding period**
Corn	61.20	60.90
Soybean meal	30.16	25.22
Fish meal	3.60	0.00
Wheat bran	0.00	10.00
Soybean oil	1.10	0.00
CaHPO_4_	1.50	1.50
Limestone	0.70	0.60
Zeolite powder	0.41	0.46
*L*-Met	0.08	0.07
NaCl	0.25	0.25
Premix	1.00	1.00

The premix for each kilogram of the diet provides the following nutrients: vitamin A (VA) 15 000 U, VD3 3300 U, VE 62.5 mg, VK 3.6 mg, VB1 3.0 mg, VB2 9.0 mg, VB6 6.0 mg, VB12 0.03 mg, folic acid 60 mg, niacin 60 mg, pantothenic acid 18 mg, Biotin 0.36 mg, choline chloride 600 mg, Se 0.15 mg, I 0.35 mg, Cu 12 mg, Mn 60 mg, Fe 80 mg, Zn 75 mg, and other additives such as antibacterial growth promoting agents and antioxidants.

**Table 2:** Nutrient levels of the basal rations.

**Nutrient components**	**Brooding period**	**Breeding period**
Metabolizable energy/(MJ/kg)	12.13	11.63
Crude protein (%)	19.30	15.50
Lysine (%)	0.98	0.79
Methionine (%)	0.08	0.07
Calcium (%)	0.85	0.80
Available phosphorus (%)	0.37	0.37

The metabolizable energy was calculated, while the remaining portion was quantified.

### 2.3 Sequencing of small RNA (sRNA)

#### 2.3.1 RNA isolation, sRNA library construction, and sequencing

The Trizol reagent was used for RNA extraction. Agarose gel electrophoresis was used to check RNA integrity and contamination. Nanodrop instrument was used to check RNA purity. The quantity of the RNA was quantified using Qubit and the quality of RNA was assessed using the Agilent 2100 system. The sRNA Sample Pre Kit was used to construct the sRNA library from abdominal fat tissue following the assessment of quality. This technique gained its basis through the exclusive structures at the 3′ and 5′ end of sRNA. Onto these ends are attached a complete phosphate group at the 5′ end and a hydroxyl group at the 3′ end. The process started with the extraction of total RNA. After that, adapters were ligated to both ends of the sRNA and reverse transcribed to cDNA. After the PCR amplification phase, the target DNA fragments were separated using PAGE gel electrophoresis. The gel was then excised to obtain the cDNA library. Once the library construction was completed, an initial quantification was performed using a Qubit 2.0 device. The library was diluted to a concentration of 1 ng/μl. Following this, the size of the insert was assessed with an Agilent 2100 system (Agilent Technologies, CA, United States). Finally, the effective concentration was determined via RT-qPCR, ensuring that a satisfactory effective concentration exceeded 2 nM. Libraries meeting the quality standards were subsequently sequenced by Beijing Novogene Technology Co., Ltd. employing the Illumina SE50 technique.

#### 2.3.2 Quality control of sRNA sequencing and reference genome alignment

Raw sequencing reads underwent processing to evaluate sequencing quality, determine the length distribution of sRNA reads, and eliminate sequences exhibiting more than 10% N content, low-quality sequences, those containing 5′ adapters, sequences lacking 3′ adapters or insertion fragments, as well as sequences with polyA/T/G/C. Clean reads within the range of 18–35 nt were subsequently employed in the ensuing analyses.

The Bowtie (v0.12.9) software was used to align length-filtered clean reads with the chicken genome, facilitating the annotation of various RNA types, including rRNA, tRNA, snRNA, snoRNA, and repetitive sequences. Subsequently, the remaining sequences were compared with the chicken miRBase database to identify known miRNAs. At the same time, the unannotated sequences were aligned with the chicken genome sequence to uncover potential novel miRNAs. To predict the hairpin structures and folding energies of these sequences, MiREvo (v1.1) and mirdeep2 (v2.0.0.5) were utilized. Only sequences that exhibited stem-loop hairpin structures were considered as potential novel miRNAs. Ultimately, we retained high-confidence candidate miRNAs that had a miRDeep2 composite score >4 and were supported by all structural and evolutionary filters in the miREvo analysis.

#### 2.3.3 Analysis of differential miRNA expression

For the differential expression analysis of miRNAs, the expression levels of both known and novel miRNAs were statistically evaluated, and normalization was performed using the Transcripts Per Million (TPM) method. The normalized expression values were calculated utilizing the formula: (read count × 1,000,000)/total miRNA read count in the library. Differentially expressed miRNAs were identified through the DESeq2 (v1.20.0) software, which conducts pairwise sample analysis based on a negative binomial distribution, applying a threshold of *P*-value < 0.05 and | log2(fold change) | >1.

#### 2.3.4 Differentially expressed miRNA target gene prediction, gene ontology (GO) and Kyoto encyclopedia of genes and genomes (KEGG) enrichment analysis

The prediction of target genes for differentially expressed miRNAs was achieved using both miRanda (v2.0.0.8) and RNAhybrid (v2.1.2) software, with the intersection of results taken into consideration. To investigate the functional characteristics of target genes, we performed GO enrichment analysis using GOSeq/topGO (Release 2.12). Meanwhile, pathway enrichment analysis was conducted with KOBAS (v2.0) based on the KEGG database to identify significantly associated pathways. In the analysis, the Benjamini–Hochberg (BH) method was applied for multiple testing correction of *P*-values in enrichment analysis. Pathways or functional terms with adjusted *P*-values < 0.05 were considered significantly enriched.

#### 2.3.5 sRNA sequencing analysis

This study employed standardized statistical methods and software for data analysis rigor for its data analysis. Following the quality assessment of raw sequencing data, the sequences containing >10% *N* bases, low-quality reads, adapter-contaminated reads, and polyA/T/G/C sequences were filtered out. Meanwhile, the clean reads of lengths 18–35 nt were reserved for the further analysis. The sequences were aligned to the chicken reference genome using Bowtie (v0.12.9), followed by comprehensive RNA annotation. Known miRNAs were identified by aligning with the miRBase database, while novel miRNAs were predicted using both MiREvo (v1.1) and mirdeep2 (v2.0.0.5) software, with only sequences exhibiting typical stem-loop structures being retained as candidates.

miRNA expression levels were normalized using transcripts per million (TPM) method. Differential expression analysis was performed using DESeq2 (v1.20.0) software, with screening criteria set at adjusted *P*-value < 0.05 and | log2(fold change) | >1. For the prediction of target genes of differentially expressed miRNAs, the results from both miRanda (v2.0.0.8) and RNAhybrid (v2.1.2) software were integrated, with their intersection taken as the final predicted target genes. Subsequently, GOSeq/topGO (Release 2.12) was employed for GO enrichment analysis, and KOBAS (v2.0) was used for KEGG pathway enrichment analysis. All enrichment analysis results were adjusted for *P*-values using the Benjamini–Hochberg (BH) method, with an adjusted *P*-value < 0.05 considered statistically significant.

### 2.4 Transcriptome sequencing

#### 2.4.1 Total RNA extraction and transcriptome sequencing of abdominal fat tissue

In terms of transcriptome sequencing, total RNA was extracted from abdominal fat tissue using TRIzol Reagent. The concentration and purity of RNA were assessed via a NanoDrop 2000 spectrophotometer, and RNA integrity was precisely evaluated using the RNA Nano 6000 detection kit from the Agilent Bioanalyzer 2100. After confirming the quality of the RNA, a cDNA library was generated, followed by sequencing on the Illumina NovaSeq 6000 platform in PE150 configuration. The sequencing was conducted by Beijing Novogene Technology Co., Ltd. Clean data, effectively filtered to remove sequences with adapters, poly-*N* sequences, and low-quality reads (wherein reads displayed more than 10% *N* or where over 50% of the bases had a quality score of *Q* ≤ 10), were acquired. The resulting clean reads were then aligned to the reference genome utilizing the HISAT2 (v2.0.5) software. Following this, StringTie (v1.3.3b) was used to assemble and reconstruct the aligned reads. Ultimately, gene expression levels were computed through the Fragments Per Kilobase of transcript per Million mapped reads (FPKM) method.

#### 2.4.2 Differential expression gene screening

Based on the alignment results, raw read counts mapped to each gene were quantified using the featureCounts (v1.5.0p3). The DESeq2 (v1.20.0) package was used for the differential expression analysis of the data with raw count matrix as input. The first step of the software is that it assesses the raw count data by estimating size factors to normalize for library size. The second step is fitting a negative binomial distribution model, which will estimate dispersion at the gene level. The Benjamini and Hochberg method was applied to control the false discovery rate, yielding adjusted *P*-values. The criteria of |log2FC| > 1 and *P* ≤ 0.05 were used to filter differentially expressed genes (DEGs), and visualizations of the differences between the two groups were created using heatmaps and volcano plots.

#### 2.4.3 Differential expression genes GO and KEGG pathway enrichment analysis

The analysis of GO enrichment for DEGs was ultimately conducted using the clusterProfiler (v3.8.1) package. The Wallenius non-central hypergeometric distribution was used in the assessment to correct for gene length bias in DEGs. Alongside clusterProfiler (v3.8.1), the KOBAS (v2.0) database facilitated the KEGG enrichment analysis of DEGs.

#### 2.4.4 Transcriptome sequencing analysis

This study employed standardized procedures for transcriptome data analysis. Quality-controlled sequencing data were aligned to the reference genome using HISAT2 (v2.0.5) and assembled via StringTie (v1.3.3b). Gene expression levels were quantified as FPKM values. Differential expression analysis was performed based on the raw count matrix generated by featureCounts (v1.5.0p3) using DESeq2 (v1.20.0), which executed library size normalization, negative binomial model fitting, and dispersion estimation. The Benjamini–Hochberg method was applied for *P*-value adjustment to control the false discovery rate. The screening criteria for DEGs were |log2FC| > 1 and adjusted *P* ≤ 0.05. GO enrichment analysis was performed using clusterProfiler (v3.8.1) software with correction for gene length bias; KEGG pathway enrichment analysis was conducted using the KOBAS (v2.0) database, and the results were visualized through heatmaps and volcano plots.

### 2.5 RT-qPCR verification of differential expression mRNA and miRNA

To confirm the differentially expressed mRNA and miRNA, RT-qPCR techniques were applied. Total RNA was extracted from adipose tissue samples employing TRIzol. The identified differentially expressed miRNAs were reliable, we selected five miRNAs for RT-qPCR validation alongside six DEGs. The primers for mRNA were designed utilizing Prime 5.0 software (Table 3), whereas those for miRNA were developed through miRprimer2 software (Table 4). The synthesis of these primers was conducted by Kunming Qingke Biotechnology Co., Ltd. For the quantification of relative expression levels, GAPDH and U6 served as internal controls for DEMs and DEGs, respectively.

**Table 3:** Primer sequences of mRNAs used for RT-qPCR.

**Accession number**	**Gene name**	**Primer sequence (5′3′)**	**Length (bp)**
NM_204305.1	GAPDH	F: CTGGGGCTCATCTGAAGGGT	308
		R: GGACGCTGGGATGATGTTCT	
NM_204272.2	RPL39L	F: ATGTCGTCTCACAAGACCTTCAAGA	263
		R: CAGCACTACAGAAGGAATGACATGA	
NM_001012545.2	GPM6B	F: TTTTCCTCACCTACGTGCTTGG	138
		R: CTCCTGGAACCGTCATGTTTATC	
XM_046901266.1	MORN3	F: GGACGATGTTCTACCCCAGC	103
		R: TCATAGGTGGACCCGTCTCC	
NM_001031305.2	SLC9A9	F: TATGGGATCCTCTTATGCAGTTGTC	144
		R: ATACCTGTGAGACCAGCAGCTTC	
NM_001199595.2	PHLDA2	F: CAAGGAGATTGACTTTCGGTGC	85
		R: CCTCTTGTTCTGGAAGTCGATG	
XM_040696392.2	CTHRC1	F: ATAGCGGAATGTACGTTCACAAAGA	249
		R: GTTGATCCCTTCACACAGACCTT	

**Table 4:** Primer sequences of miRNAs used for the stem-loop RT-qPCR.

**Primer name**	**Primer sequence(5′3′)**	**Length (bp)**
U6	F: CTCGCTTCGGCAGCACA	94
	R: AACGCTTCACGAATTTGCGT	
gga-miR-455-3p	Loop: CTCAACTGGTGTCGTGGAGTCGGCAATTCAGTTGAGGTGTATAT	66
	F: ACACTCCAGCTGGGTGCAGTCCATGGGCAT	
	R: TGGTGTCGTGGAGTCG	
gga-miR-183	Loop: CTCAACTGGTGTCGTGGAGTCGGCAATTCAGTTGAGCAGTGAAT	
	F: ACACTCCAGCTGGGTATGGCACTGGTAGAAT	67
	R: TGGTGTCGTGGAGTCG	
gga-miR-17-5p	Loop: CTCAACTGGTGTCGTGGAGTCGGCAATTCAGTTGAGACTACCTG	68
	F:ACACTCCAGCTGGGCAAAGTGCTTACAGTGCA	
	R: TGGTGTCGTGGAGTCG	
gga-miR-9-5p	Loop: CTCAACTGGTGTCGTGGAGTCGGCAATTCAGTTGAGTCATACAG	67
	F: ACACTCCAGCTGGGTCTTTGGTTATCTAGCT	
	R: TGGTGTCGTGGAGTCG	
gga-miR-129-5p	Loop: CTCAACTGGTGTCGTGGAGTCGGCAATTCAGTTGAGGCAAGCCC	65
	F: ACACTCCAGCTGGGCTTTTTGCGGTCTGG	
	R: TGGTGTCGTGGAGTCG	

The mRNA quantitative fluorescence reaction total volume was set to 20 μl. The solution had 12.5 μl of (2 × ) TB Green Premix Ex Taq II (Tli RNase H Plus), 1 μl each of upstream and downstream primers, 2 μl of cDNA and 8.5 μl of double-distilled water (ddH_2_O). The initial step was denaturation at five degrees Celsius for 5 min after forty cycles. Every cycle consists of a denaturation step at 95 °C for 15 s and heating at 60 °C for 45 s.

Conversely, the quantitative fluorescence reaction system for miRNA was established with an overall volume of 25 μl. This configuration included 12.5 μl of 2 × TB Green Premix Taq II, 0.5 μl of a primer specific to miRNA, 0.5 μl of the mR3′ Primer; 2.5 μl of cDNA, and 9 μl of RNase-free ddH_2_O. The reaction protocol began with 15 s of pre-denaturation at 95 °C, followed by 5 s of denaturation at 95 °C, and 20 seconds of annealing at 60 °C. The said sequence was similar to that of 40 cycles. To ensure that the results were accurate, three biological replicates were done for each sample.

## 3 Results

### 3.1 Quality control of sRNA sequencing data

The quality assessment of the six samples confirmed their suitability for constructing sRNA libraries and further experimentation. After sequencing, the raw data went through quality control performed by custom scripts developed in Perl and Python. The initial read counts spanned from 13,390,828 to 15,505,114. After quality control, the valid read counts ranged from 13,032,440 to 15,515,785. All the samples displayed Q20 scores more than 98% while the Q30 scores were above 96%. The GC content for each sample was between 48.90 and 53.22%. The acquisition of high-quality sequencing data, coupled with consistent GC content, indicates that the sequencing quality is adequate for further analysis. Detailed quality control metrics for each sample can be found in Table 5.

**Table 5:** Summary of the quality of sequencing data output.

**Sample**	**Total reads**	**Clean reads**	**Q20**	**Q30**	**GC content**
CK_1	13,390,828	13,032,440 (97.32%)	98.96%	96.28%	50.47%
CK_2	14,903,068	14,607,373 (98.02%)	98.97%	96.17%	53.22%
CK_3	13,935,469	13,675,100 (98.13%)	99.06%	96.81%	49.28%
GAA_1	15,249,332	14,994,452 (98.33%)	99.06%	96.63%	49.87%
GAA_2	15,788,637	15,515,785 (98.27%)	99.57%	98.27%	48.90%
GAA_3	15,505,114	15,288,859 (98.61%)	99.56%	98.07%	50.92%

Sample is the sample id; reads is statistical raw sequence data; bases were the number of sequencing sequences multiplied by the length of sequencing sequences and converted to G; Error rate is the sequencing error rate; Q20 was the percentage of bases with Phred value greater than 20 in the total base; Q30 is the percentage of bases with Phred value greater than 30 in the total base; GC content is the percentage of the total number of bases G and C combined to calculate the total number of bases.

### 3.2 Length distribution of sRNAs

**Figure 1:**
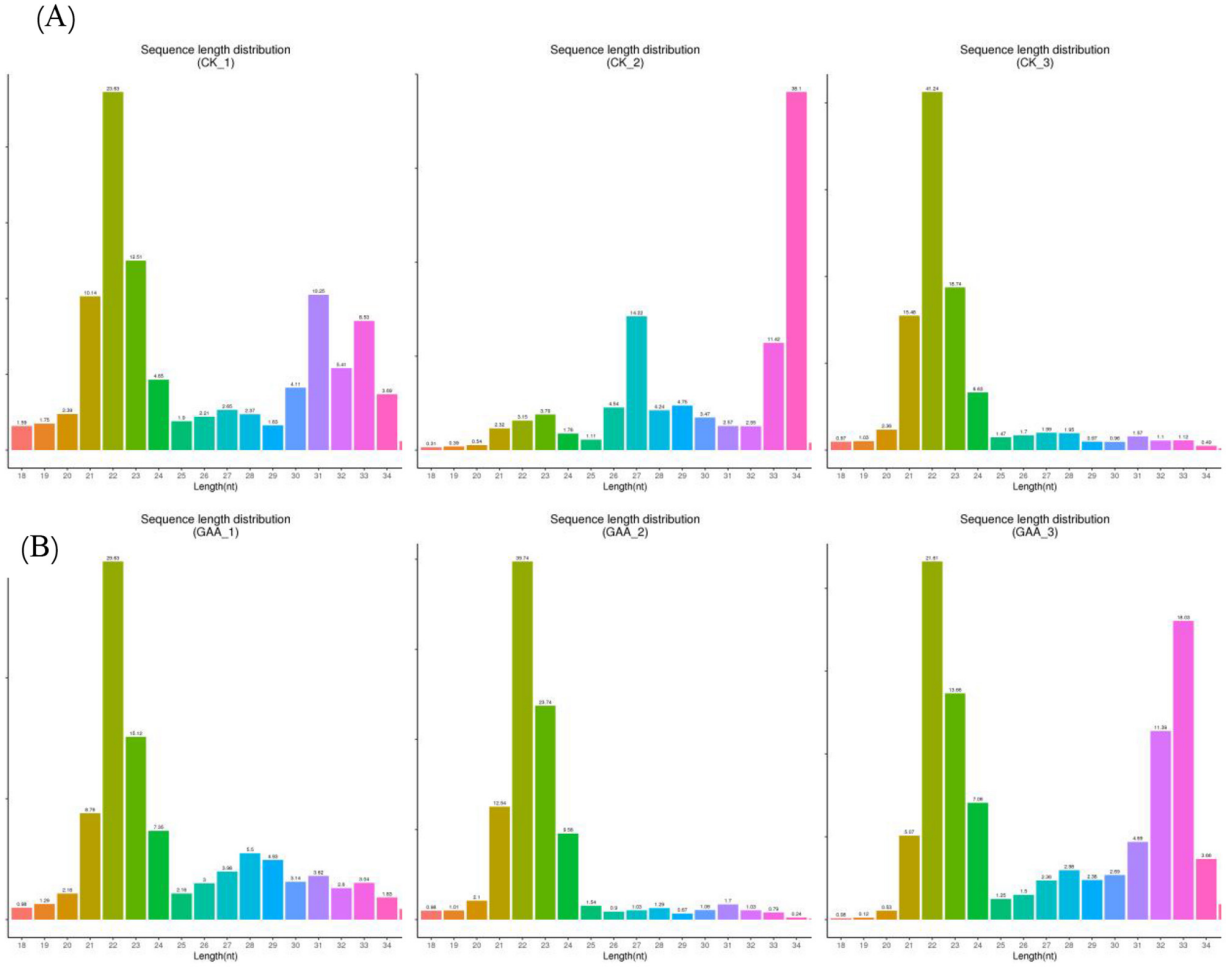
The length distribution statistics of total sRNA fragments were obtained. “CK” represents the broilers fed the basal diet; “GAA” represents the broilers fed the diet with 1.2 g/kg GAA; **(A)** is the total sRNA fragment length distribution obtained from the three samples in the control group; **(B)** is the total sRNA fragment length distribution obtained from the three samples in the 1.2 g/kg GAA group.

With high-throughput sequencing technology, we can sequence and quantitatively assess the expression of all the sRNA families present in the sample. Therefore, it is possible to analyze upstream of miRNAs, siRNAs, piRNAs and other non-coding RNAs and their target sequences. The clean reads recovered led to the selection of sRNAs with a size ranging from 18 to 35 nucleotides (Table 6). The sequenced sRNAs ranged between 20 and 24 nucleotides (Figure 1), which were Dicer products. The sRNAs show a peak at 22 nucleotides, which is also the length class for animal miRNAs.

**Table 6:** The number and types of sRNA after length screening.

**Sample**	**Total reads**	**Total bases (bp)**	**Uniq reads**	**Uniq bases (bp)**
CK_1	11,972,029	308,127,458	523,612	13,007,621
CK_2	14,223,118	429,836,924	479,679	12,434,120
CK_3	13,108,789	301,296,434	403,603	9,710,912
GAA_1	14,429,382	355,733,707	921,892	23,609,083
GAA_2	15,028,789	344,406,103	350,208	8,305,202
GAA_3	14,746,635	399,498,360	308,054	8,468,730

Sample is the sample id; total reads is the total number of sRNAs; total bases (bp) is the total length of the sRNA; uniq reads is the type of sRNA; uniq bases (bp) are the total length of each sRNA.

### 3.3 Genome alignment of sRNAs

Using Bowtie (v0.12.9), the filtered sRNAs were subjected to the complete sequence alignment to a reference genome. As per the results, a significantly high proportion of clean reads were effectively mapped on the reference genome. The control group had an observed value of 88.27%. By contrast, the mapping rate for the GAA group was slightly lower (86.33%). The average percentages of reads aligning in the same orientation to the reference sequence were 56.64% and 50.70%, respectively, while those aligning in the opposite direction averaged 31.63% and 35.62%. Table 7 summarizes the complete information regarding the alignment of samples.

**Table 7:** Comparisons with reference sequences.

**Sample**	**Total sRNA**	**Mapped sRNA**	**+ Mapped sRNA**	**– Mapped sRNA**
CK_1	11,972,029 (100.00%)	1,0476,888 (87.51%)	6,161,716 (51.47%)	4,315,172 (36.04%)
CK_2	14,223,118 (100.00%)	12,029,114 (84.57%)	10,995,417 (77.31%)	1,033,697 (7.27%)
CK_3	13,108,789 (100.00%)	12,154,329 (92.72%)	5,393,263 (41.14%)	6,761,066 (51.58%)
GAA_1	14,429,382 (100.00%)	11,599,574 (80.39%)	6,959,149 (48.23%)	4,640,425 (32.16%)
GAA_2	15,028,789 (100.00%)	14,148,598 (94.14%)	6,889,187 (45.84%)	7,259,411 (48.30%)
GAA_3	14,746,635 (100.00%)	12,453,480 (84.45%)	8,558,301 (58.04%)	3,895,179 (26.41%)

Sample is the sample id; total sRNA, clean reads of each sample obtained after length screening; mapped sRNA, the number and percentage of clean reads mapped to the reference sequence in the sample; +mapped sRNA, the number and percentage of reads in the clean reads of the sample that are mapped to the same direction of the reference sequence; –mapped sRNA, the number and percentage of reads in the clean reads of the sample that are mapped to the chain in the opposite direction of the reference sequence.

### 3.4 Screening of differentially expressed miRNAs and RT-qPCR validation

**Figure 2:**
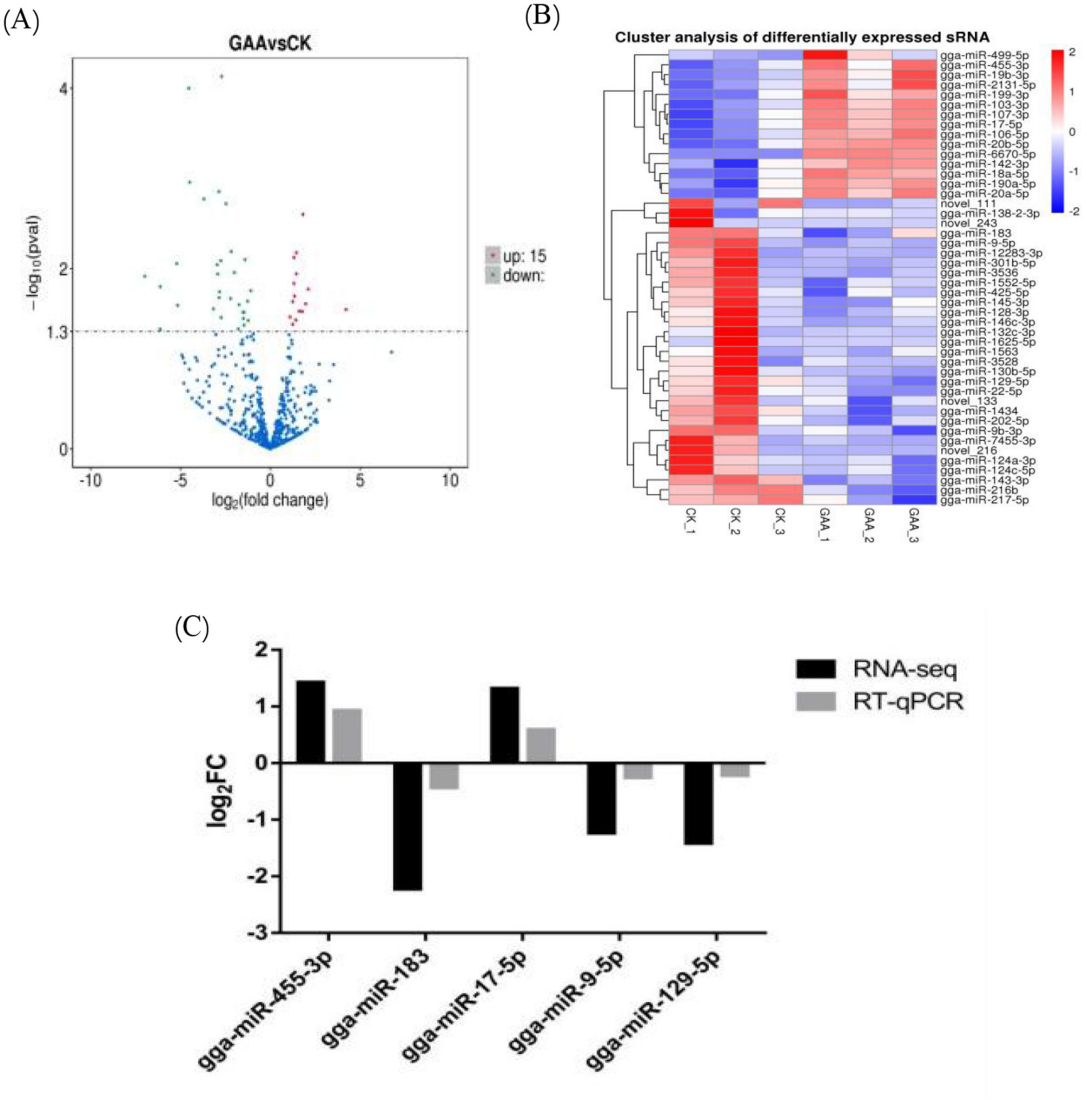
**(A)** Differentially expressed miRNA volcano map for GAA group vs. control group in sRNA sequencing analysis; **(B)** differentially expressed miRNA cluster diagram; **(C)** illustration of RT-qPCR confirmation results for five selected differentially expressed miRNAs.

In order to significantly compare the specimens, the expression levels of the known miRNAs and the newly identified miRNAs in each specimen were measured. To ensure consistency and reliability in the analysis, these expression levels were normalized using a metric known as transcripts per million (TPM). Pairwise analysis was performed using DESeq2 (v1.20.0) with negative binomial distribution, as shown in the volcano plot in Figure 2A. According to the volcano plot, the expression level of 46 miRNAs is significantly different between the control groups and GAA. Fifteen miRNAs showed upregulation while 31 miRNAs showed downregulation. The validity of these differentially expressed miRNAs requires further investigation.

The expression levels of miRNAs were influenced by GAA. A hierarchical clustering analysis on differentially expressed miRNAs across the six samples (Figure 2B) classified the six samples into two major branches, indicating high consistency among the samples and hence strength of the experimental design and analysis. Among the various miRNAs that show differential expression, a few have been identified as significant to fat deposition, particularly gga-miR-455-3p and gga-miR-425-5p. A subset of five differentially expressed miRNAs was randomly chosen for validation via reverse transcription quantitative polymerase chain reaction (RT-qPCR), with results that closely matched those obtained from sRNA sequencing (Figure 2C). This concordance emphasizes the consistency and reliability of the sRNA sequencing data, thereby affirming its appropriateness for further analytical endeavors.

### 3.5 Prediction and bioinformatics analysis of miRNA target genes with differential expression

In the following section, titled Target Gene Prediction for Differentially Expressed miRNAs and Bioinformatics Analysis, the prediction of target genes for the identified differentially expressed miRNAs was performed by analyzing their interactions with the help of miRanda (v2.0.0.8) and RNAhybrid (v2.1.2) software. This analysis enabled the discovery of target genes linked to the miRNAs that were expressed differently. The results indicated that one miRNA has the potential to control numerous genes, while on the other hand, a singular gene can be influenced by multiple miRNAs. Interestingly, the miRNAs predicted to have the most significant regulatory influence on target genes were gga-miR-1625-5p (which regulates 251 genes), gga-miR-7455-3p (influencing 196 genes), gga-miR-107-3p (affecting 175 genes), gga-miR-103-3p (impacting 161 genes), gga-miR-12283-3p (regulating 111 genes), gga-miR-2131-5p (which regulates 91 genes), gga-miR-3528 (overseeing 83 genes), gga-miR-455-3p (controlling 77 genes), and gga-miR-130b-5p (regulating 63 genes).

**Figure 3:**
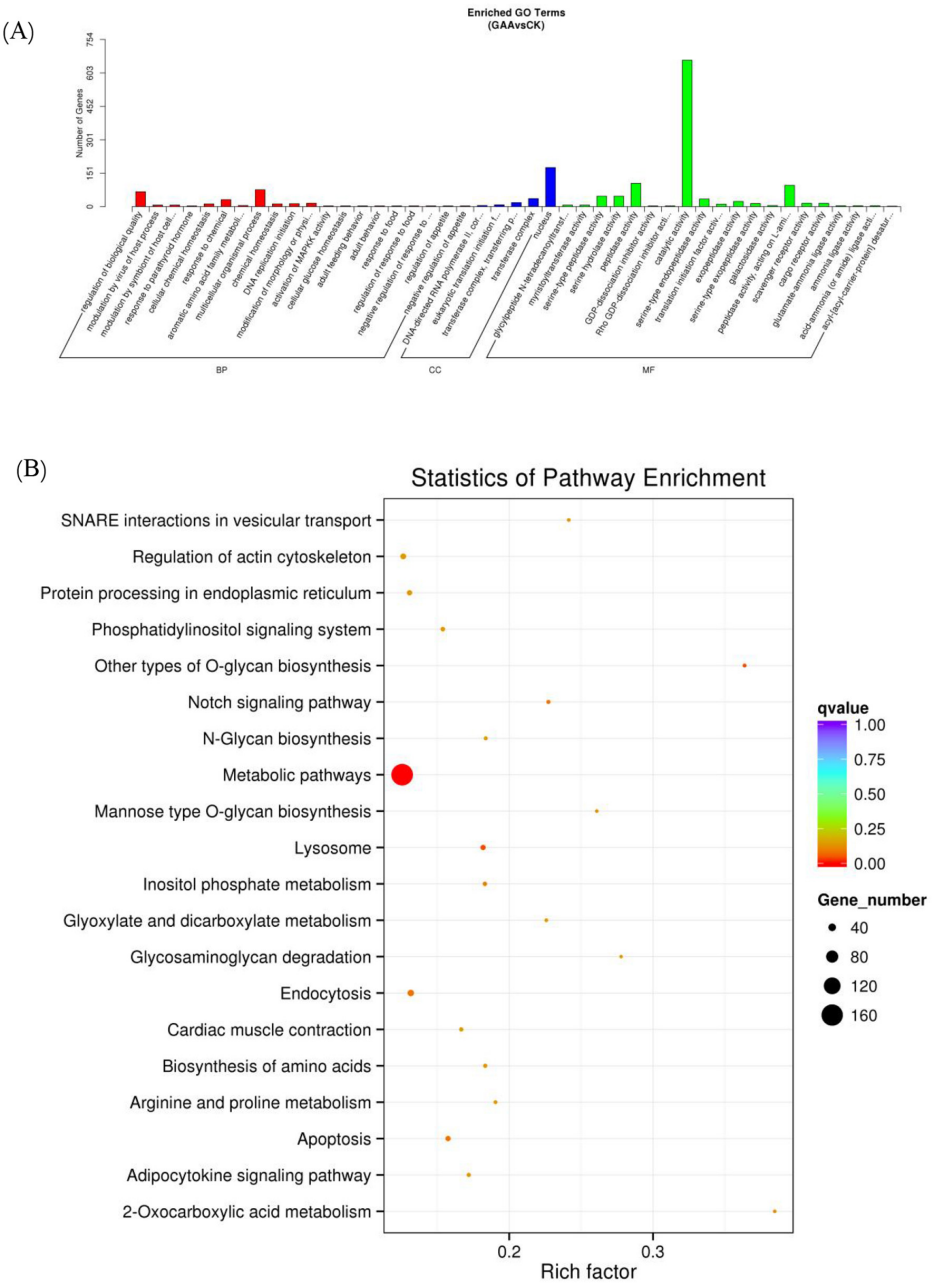
GO enrichment column **(A)** and KEGG enrichment scatter plot **(B)** of candidate target genes.

Furthermore, enrichment analyses were conducted utilizing GO and the KEGG on gene sets linked to the differentially expressed miRNAs (see Figures 3A, B). The findings from the GO enrichment analysis indicated that the target genes in the GAA group showed a marked enrichment in processes related to catalytic activities, cellular nuclear functions, and peptidase activity when compared to the control group. Similarly, the KEGG enrichment assay indicated that the target genes in the GAA group were significantly enriched in metabolic processes, regulation of actin cytoskeleton, endocytosis, lysosome functions, apoptosis, and protein processing in the endoplasmic reticulum.

Lipid metabolic pathways were highly enriched in genes with member functions. Lipid synthesis (lipogenesis) and lipid breakdown (lipolysis) are the main metabolic processes. The synthesis and proper folding of enzymes and membrane proteins that are necessary for lipid synthesis requires protein processing in the endoplasmic reticulum. Moreover, the actin cytoskeleton was regulated, which is important to adipocyte differentiation and lipid droplet dynamics, playing a pivotal role in adipose tissue expansion. Lipid mobilization and turnover pathways were also significantly enriched. Functions of lysosome directly take part in lipid degradation from lipophagy. Endocytosis can modulate lipid signaling and uptake by regulating membrane receptors and transporters. Moreover, apoptosis is involved in adipose tissue remodeling and may influence overall lipid homeostasis indirectly. Together, these enriched pathways suggest that GAA treatment may modulate the molecular machinery controlling fat deposition and degradation.

### 3.6 Transcriptome analysis

**Figure 4:**
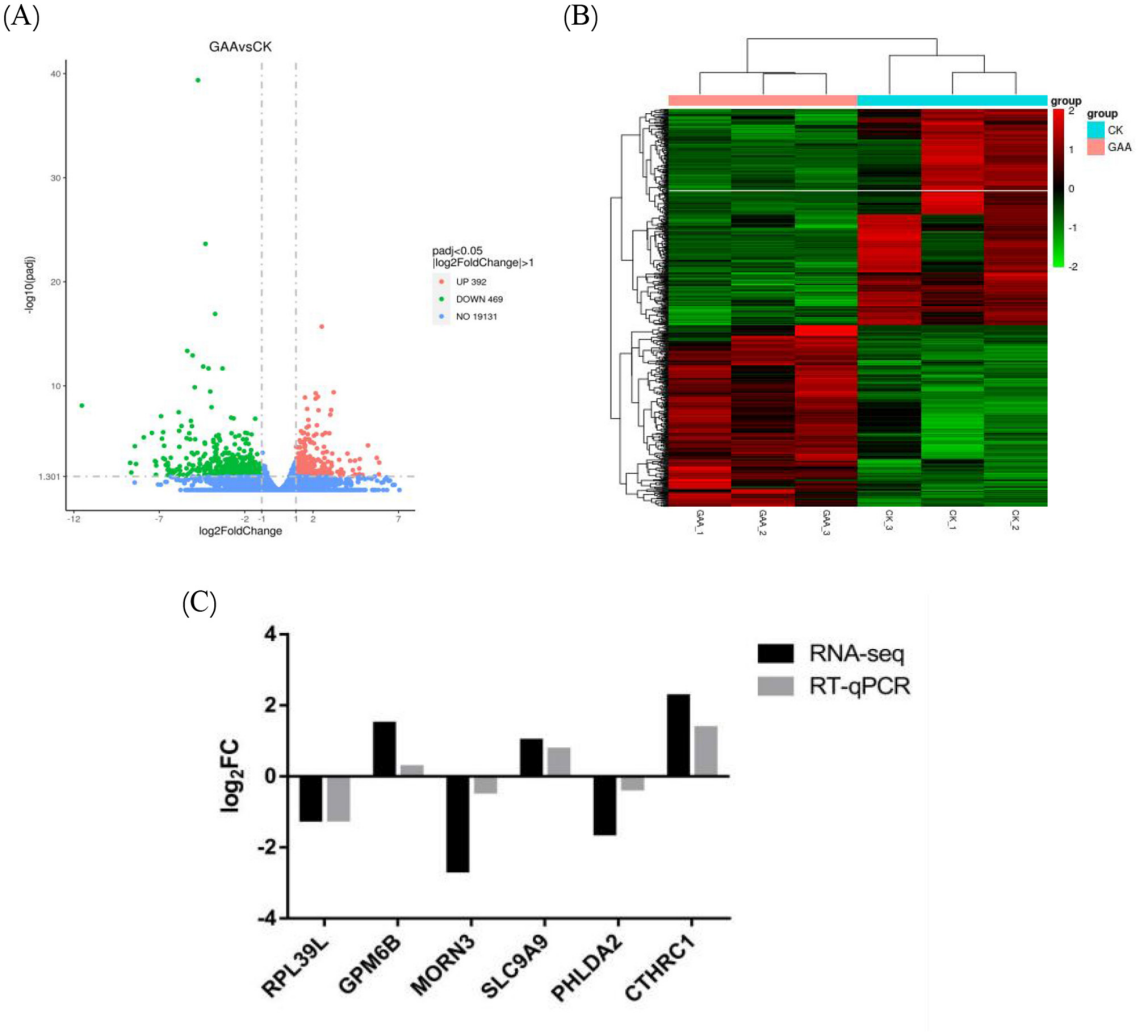
**(A)** Differentially expressed mRNA volcano map for GAA group vs. control group in sRNA sequencing analysis; **(B)** differentially expressed mRNA cluster diagram; **(C)** illustration of RT-qPCR confirmation results for six selected differentially expressed mRNAs.

The expression levels of known and novel mRNAs were quantified and normalized across each sample using Transcripts Per Million (TPM) in the Transcriptome Analysis section. Using DESeq2 (version 1.20.0) and the negative binomial distribution, we performed sample pairwise analysis which resulted in a volcano plot shown in Figure 4A. The volcano plot reveals that 861 mRNAs significantly differed in expression between the GAA group and the control group, with 392 mRNAs upregulated and 469 mRNAs downregulated. To investigate the relationship of GAA with the mRNA expression levels, we performed a hierarchical clustering analysis of differentially expressed mRNAs from the six samples (Figure 4B). This analysis effectively separated the six samples into two main branches, which indicated strong sample consistency and a sound study design. Fat deposition-related mRNAs were both RPL39L and MORN3 among differentially expressed mRNAs. Four randomly selected differentially expressed mRNAs were validated using RT-qPCR, and the results were consistent with transcriptome sequencing data (Figure 4C), which further verified the reproducibility and reliability of the transcriptome sequencing data.

**Figure 5:**
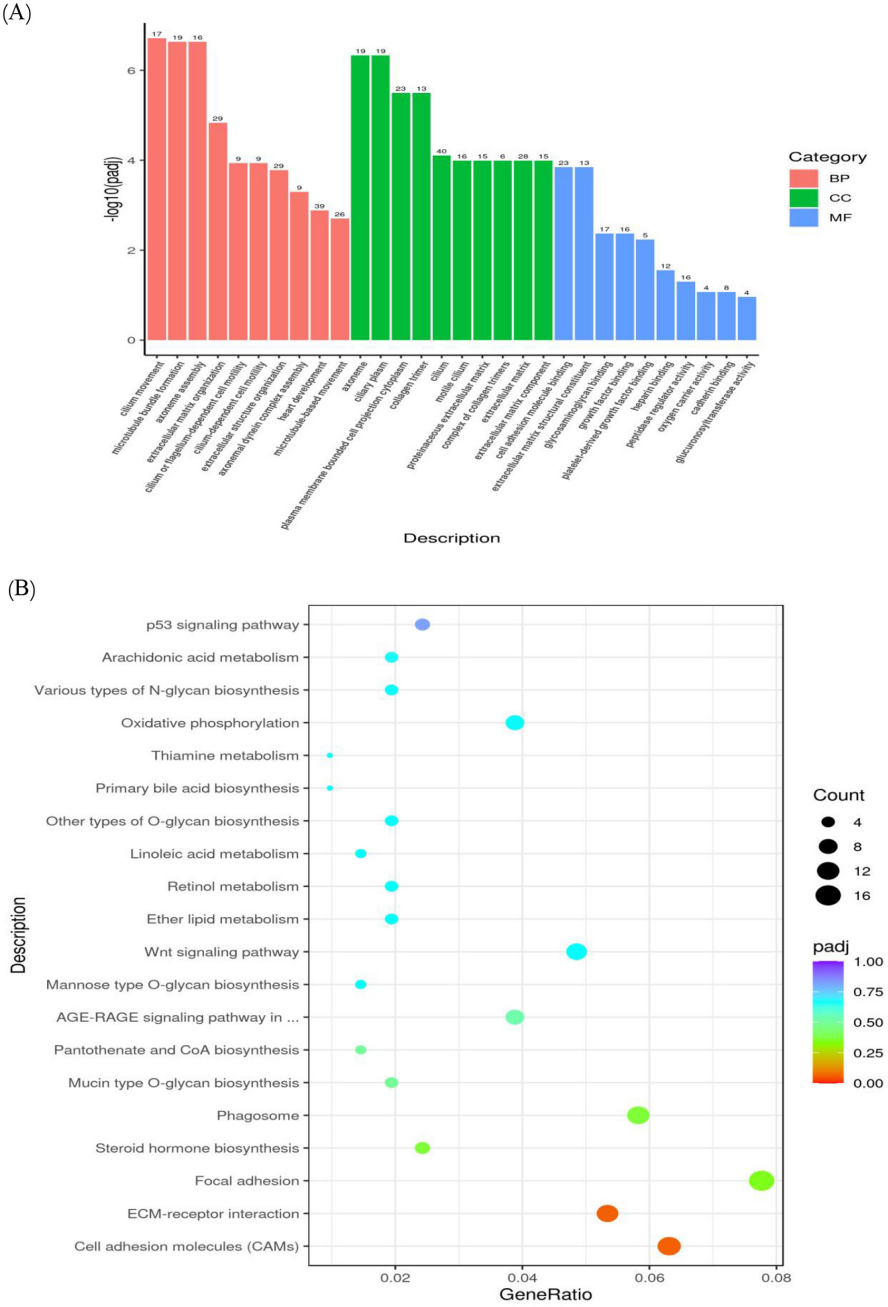
GO enrichment column **(A)** and KEGG enrichment scatter plot **(B)** of differentially expressed mRNA.

Therefore, enrichment analyses for GO and KEGG were performed for the DEGs (Figures 5A, B). According to the GO enrichment assessment, compared to control genes, GAA genes mainly have extracellular matrix-related functions.

The KEGG enrichment analysis on DEGs reveals that, compared to control, the GAA group shows primary enrichment for several categories. These included CAMs, focal adhesion, synthesis of steroid hormones, phagosome dynamics, biosynthesis of mucin-type O-glycans and synthesis of pantothenic acid and coenzyme A. In addition, the analysis analyzed the AGE-RAGE signaling pathway with respect to complications arising from diabetes.

Focal adhesions and cell adhesion molecules (CAMs) play a role in adipocyte differentiation, lipid droplet formation, and overall adipose tissue expansion and remodeling. The AGE-RAGE signaling pathway acts as a crucial pro-inflammatory and profibrotic pathway in diabetic complications. The phenomenon of enrichment suggests a potential mechanism through which GAA may impact inflammatory, fibrotic, and insulin-sensitive features of adipose tissue, leading to a concurrent regulation of lipid storage capacity (deposition) and metabolic turnover. The biosynthesis of steroid hormones, which act as potent regulators (e.g. glucocorticoids and sex hormones), is directly relevant.

### 3.7 Analysis of differential expression miRNA and mRNA regulatory networks

**Figure 6:**
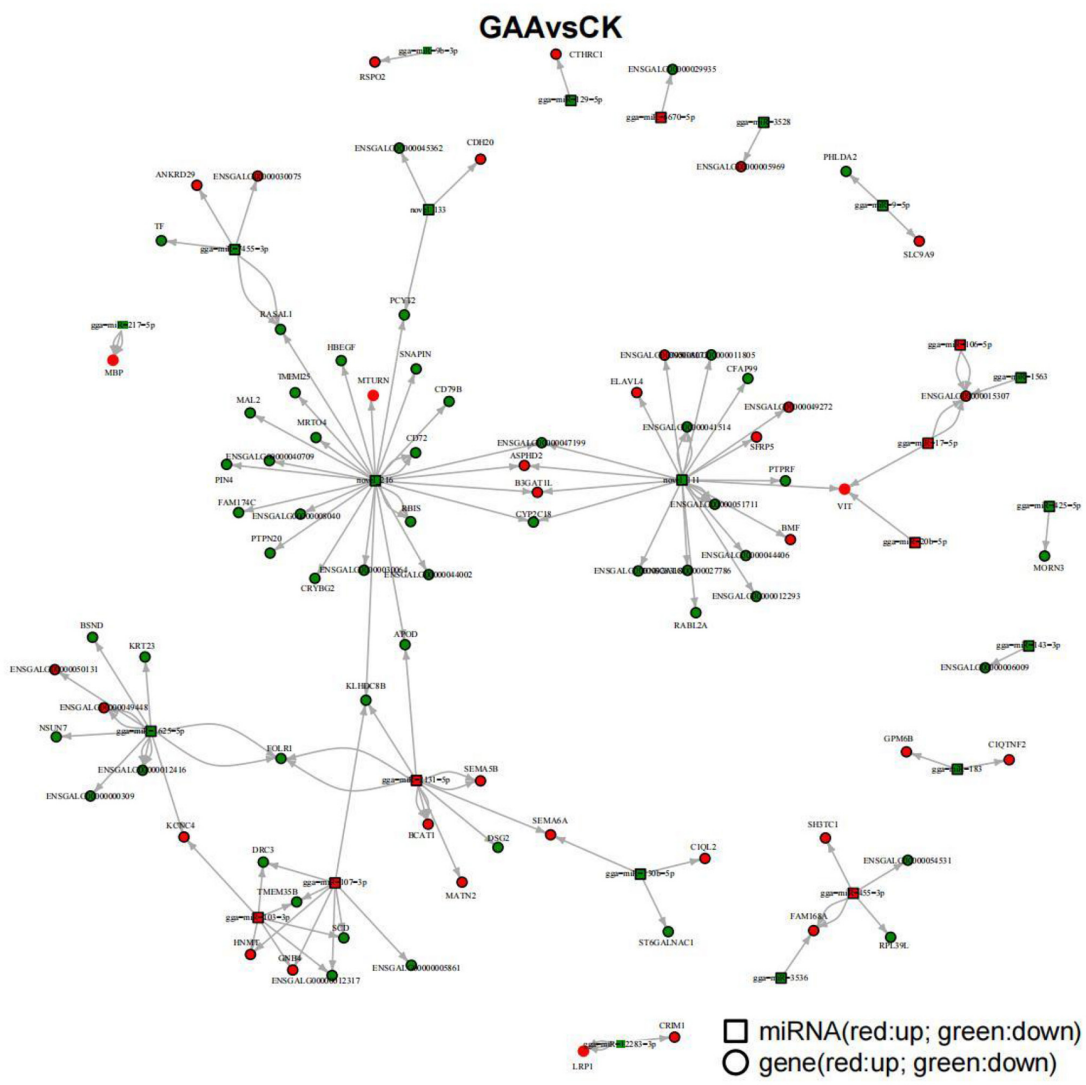
Analysis of miRNA and target gene regulation network.

To generate a network diagram using Cytoscape, we collected gene targets of differentially expressed miRNAs with results of DEGs in our regulatory network analysis of expression differentially expressed miRNAs and mRNAs. Within the networks of the GAA group and the control group (Figure 6), 25 miRNAs were linked to 86 DEGs, thereby building a complex network. This includes miRNAs such as gga-miR-9b-3p, gga-miR-129-5p, gga-miR-7455-3p, gga-miR-217-5p, gga-miR-9-5p, gga-miR-17-5p, gga-miR-20b-5p, gga-miR-425-5p, gga-miR-183, gga-miR-1625-5p, gga-miR-103-3p, gga-miR-107-3p, gga-miR-2131-5p, gga-miR-130b-5p, gga-miR-12283-3p, gga-miR-3536, gga-miR-455-3p, among others. Comprehensive details about the regulatory connections between these miRNAs and their corresponding target genes are available in Table 8.

**Table 8:** Some miRNA and target gene regulation relationship.

**miRNA**	**Target gene**
gga-miR-9b-3p ↓	Up: RSPO2
gga-miR-129-5p ↓	Down: CTHRC1
gga-miR-7455-3p ↓	Up: ANKRD29
	Down: TF, RASAL1
gga-miR-217-5p ↓	Up: MBP
gga-miR-9-5p ↓	Up: SLC9A9
	Down: PHLDA2
gga-miR-17-5p ↑	Up: VIT
gga-miR-20b-5p ↑	Up: VIT
gga-miR-425-5p ↓	Down: MORN3
gga-miR-183 ↓	Up: GPM6B, C1QTNF2
gga-miR-1625-5p ↓	Up: KCNC4
	Down: BSND, KRT23, NSUN7, FOLR1
gga-miR-103-3p ↑	Up: KCNC4, HNMT, GNB4
	Down: DRC3, TMEM35B, SCD
gga-miR-107-3p ↑	Down: DRC3, TMEM35B, SCD, KLHDC8B
gga-miR-2131-5p ↑	Up: SEMA5B, SEMA6A, MATN2, BCAT1
	Down: DSG2, FOLR1, KLHDC8B, APOD
gga-miR-130b-5p ↓	Up: SEMA6A, C1QL2
	Down: ST6GALNAC1
gga-miR-12283-3p ↓	Up: CRIM1, LRP1
gga-miR-3536 ↓	Up: FAM168A
gga-miR-455-3p ↑	Up: SH3TC1, FAM168A
	Down: RPL39L

“↑↓” indicates the upregulation or downregulation of miRNA; up indicates the upregulation of target gene, and down indicates the downregulation of target gene.

## 4 Discussion

**Figure 7:**
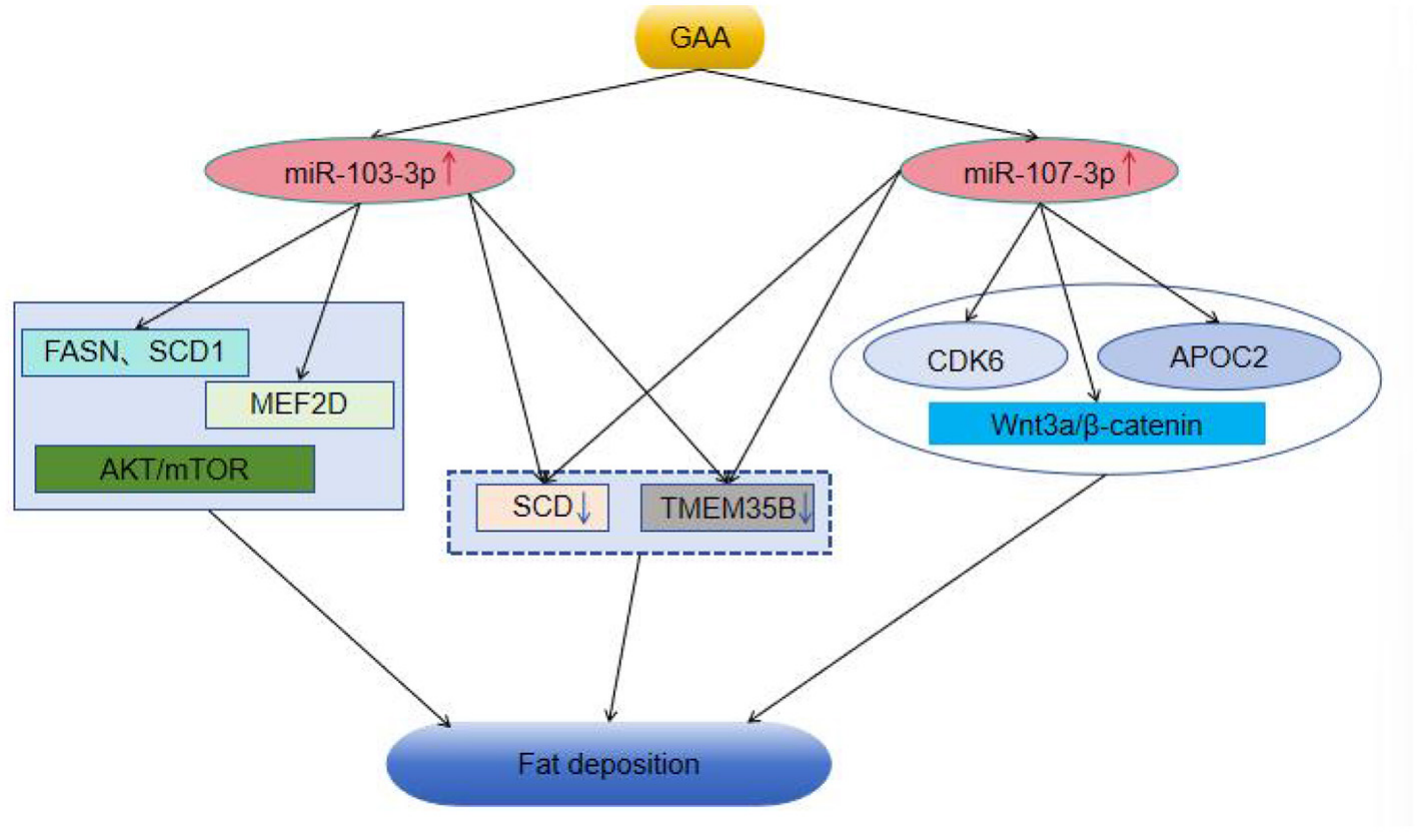
The mechanism by which GAA reduces fat deposition through miRNA-targeted gene regulatory signaling pathway. *Note* “↑↓” indicates upregulation or downregulation; an upward arrow denotes upregulation of the target gene, while a downward arrow denotes downregulation of the target gene.

Exogenous GAA administration modulates energy distribution within muscle cells (28, 29), leading to decreased activity of key metabolic enzymes and, consequently, reduced hepatic fat synthesis. Additionally, it promotes the process of β-oxidation of fatty acids, ultimately contributing to a reduction in abdominal fat accumulation in broiler chickens (11, 30, 31). This occurs via either a reduction in adipocyte size or number. Our previous study revealed that a diet supplemented with 1,200 mg/kg of GAA reduced the abdominal fat content and size of adipocytes in broiler chicken. Therefore, we propose that GAA reduces fat storage by enhancing lipid metabolism and promoting the utilization of excess energy, thereby inhibiting its conversion into adipose tissue. To visually summarize the proposed molecular pathways through which GAA may exert its anti-adipogenic effects, we have constructed a schematic model (refer to Figure 7).

To interpret these findings, we explored potential mechanistic insights from existing literature, primarily in mammalian models. TMEM is integral to muscle development and lipid metabolism. Research indicates a significant elevation in the overall level of diacylglycerol (DAG) in cells overexpressing TMEM68, demonstrating that DAG functions as an acyl receptor in the enzymatic reaction catalyzed by TMEM68 for triacylglycerol (TAG) formation, thereby highlighting TMEM8's role in regulating lipid metabolism through the catalysis of TAG synthesis (32, 33). Moreover, TMEM88 is involved in promoting lipid synthesis and cytokine secretion via the Wnt/β-catenin signaling pathway (34, 35). TMEM16F interacts with lipids, as noted by Tang et al. (36). Moreover, studies suggest that the TMEM18 gene is essential for the regulation of adipocyte differentiation via the central nervous system. According to Renström et al. (37) and Wiemerslage et al. (38), this regulation influences obesity and is implicated in the metabolic regulation of triglycerides and other lipids in adipocytes and pre-adipocytes. Although the specific function of TMEM35B in avian lipid metabolism is not yet defined, its downregulation in our study and its targeting by miRNAs suggest a potential, yet hypothetical, role in the GAA-mediated reduction of fat deposition.

Stearoyl-CoA desaturase (SCD) is a crucial fatty acid metabolism enzyme. It introduces a double bond between the ninth and tenth carbon atoms of saturated fatty acids (SFA). The process happens specifically with palmitoyl-CoA (C16:0) and stearoyl-CoA (C18:0) generating monoenoic fatty acids (MEFAs) such as palmitoleic acid (C16:1) as well as oleic acid (C18:1) (39–41). SCD actively participates in the regulation of lipid metabolism.

Lipids are essential molecules that are made and broken down by living things. Monounsaturated fatty acids (MUFA) are vital in the synthesis of complex lipids, including triglycerides (TG), phospholipids, and cholesterol esters. The synthesis of these complex lipids is crucial for maintaining the structural integrity of cellular membranes, storing lipids in fat cells, and regulating energy metabolism (42). Research indicates that SCD plays a significant role in the metabolic pathway of fatty acids and is an essential element in the metabolism of unsaturated fatty acids (43). SCD is responsible for controlling the synthesis of monounsaturated fatty acids, which subsequently influences the fluidity of membranes and the metabolism of lipids within cells (39, 44, 45).

Studies with knockout mice and the model organism *Caenorhabditis elegans* show that SCD1 knockout (or deficiency) increases SFA and decreases MUFA and shifts fatty acid metabolism from biosynthesis to oxidation (19, 46). Mice that completely lack SCD1, known as global knockout (GKO) mice, show an elevated metabolic rate due to increased lipid oxidation, decreased lipid synthesis, and increased insulin sensitivity. These factors together confer resistance to obesity and fatty liver degeneration that often accompanies high-carbohydrate and high-fat diets in these mice (19). In high-fat diet-fed mice, the SCD1 inhibitor E6446 ameliorates hepatic steatosis, lipid droplet accumulation, and insulin resistance by modulating hepatic lipid metabolism pathways (47). Therefore, the observed downregulation of SCD in our study aligns with a metabolic state that favors reduced lipid synthesis and/or increased oxidation, as seen in other models. However, its direct causal role in the context of GAA action in broilers remains to be functionally validated.

miRNAs are critical mediators of lipid synthesis, fat oxidation and lipoprotein production (48, 49). Among these, miR-103 is particularly notable for its significant role in insulin resistance and lipid metabolism. This specific microRNA inhibits *de novo* lipogenesis by targeting the fatty acid synthase gene (FASN) and SCD1 in murine liver tissues, thereby contributing to the mitigation of obesity and diet-induced fatty liver disease, as reported by Zhang et al. (50). In contrast, Holik et al. (51) in models utilizing 3T3-L1 mouse pre-adipocytes, it has been noted that miR-103 exerts a unique function by encouraging lipid accumulation. In 3T3-L1 mouse embryonic fibroblasts, miR-103-3p promotes adipocyte differentiation and lipid synthesis by inhibiting the myocyte enhancer factor 2D (MEF2D) and activating the protein kinase-B/mechanistic target of rapamycin (AKT/mTOR) signaling pathway (52, 53). When miR-103/107 is deactivated in adipocytes, noticeable changes occur, including reduced adipocyte size, decreased overall fat mass in mice, improved insulin sensitivity, and enhanced glucose uptake following insulin exposure, as indicated by Trajkovski et al. (21). During porcine adipocyte differentiation, the expression of miR-103-3p (an important subtype of miR-103) is significantly upregulated, while inhibiting its expression can effectively block the differentiation of preadipocytes (54). Additionally, Liu's study reveals that miR-103-3p plays a role in controlling the growth and lipid metabolism in epithelial cells found within bovine mammary tissue (55). Lin and associates showed that a significant rise in pre-miR-103-1 expression in GMECs, attained through adenoviral infection, markedly increases triglyceride levels and lipid droplet formation in these cells (56). Our data indicate that the upregulation of miR-103-3p is associated with the downregulation of the SCD and TMEM35B genes. However, the role of miR-103-3p as a negative regulator of SCD and TMEM35B in broiler adipocytes necessitates further experimental validation.

MiR-107 plays essential roles in a spectrum of biological processes, including the regulation of insulin sensitivity, lipid metabolism, lipogenesis, tumor angiogenesis, and cell proliferation (21, 53, 57). Importantly, miR-107 influences the rise in lipid accumulation within the liver by impacting fatty acid oxidation, as evidenced by Bhatia et al. (58, 59). Furthermore, Ahonen et al. (60) demonstrated that miR-107 promotes adipocyte differentiation and ectopic fat accumulation by interacting with cyclin-dependent kinase 6 (CDK6). Research in bovine adipose tissue has demonstrated that miR-107, which is highly expressed there, inhibits adipocyte differentiation by modulating apolipoprotein C2 (APOC2) (61). It suggests that miR-103/107 induce pre-adipocyte apoptosis specifically through endoplasmic reticulum mechanisms and by stimulating the Wnt3a/β-catenin pathway, pointing to a novel potential strategy against obesity and metabolic syndrome (62). Previous research has also identified miR-107 as a possible regulatory factor associated with abdominal fat accumulating in chickens (63). The expression of gga-miR-107-3p is negatively correlated with the levels of SCD and TMEM35B. This correlation suggests that miR-107-3p may exert the effect of GAA by suppressing SCD and TMEM35B in chicken, however, this regulatory relationship requires further verification through *in vitro* experiments in broilers.

## 5 Conclusion

This study demonstrates that dietary GAA effectively reduces abdominal fat deposition in broilers. Integrated omics analysis revealed a potential mechanism: GAA upregulates gga-miR-103-3p and gga-miR-107-3p, leading to the downregulation of key target genes, SCD and TMEM35B. Given the established role of SCD in lipogenesis and the putative role of TMEM proteins in lipid metabolism, this miR-103/107–SCD/TMEM35B axis provides a novel mechanistic explanation for the anti-adipogenic effect of GAA. Although this study has the above new findings, it still has the following limitations, including the sample size (three biological replicates per group) and the correlative nature of the omics data; thus, the proposed regulatory pathway requires further direct functional validation in avian models. Collectively, our work identifies a possible molecular pathway and provides a strong theoretical foundation for using GAA as a feed additive. These results suggest that GAA supplementation could be a viable strategy to enhance lean meat yield and improve carcass quality in commercial broiler production, potentially offering economic benefits through improved feed efficiency and product value.

## Data Availability

The raw data of transcriptome presented in the study are deposited in the Sequence Read Archive repository, accession number PRJNA1381682. The raw data of Small RNA transcriptome presented in the study are deposited in the Sequence Read Archive repository, accession number PRJNA1382004.
